# Complete Genome Sequence of Bifidobacterium animalis subsp. *lactis* BLa80, a Strain Isolated from Human Breast Milk

**DOI:** 10.1128/mra.00465-22

**Published:** 2023-05-22

**Authors:** Mingming Zhu, Jianguo Zhu, Shuguang Fang, Bin Zhao

**Affiliations:** a Wuhan Wecare Probiotic Research Institute, Wuhan, China; b Wecare Probiotics Co., Ltd., Suzhou, China; c State Key Laboratory of Agricultural Microbiology, Huazhong Agricultural University, Wuhan, China; Loyola University Chicago

## Abstract

We report the complete genome sequence of Bifidobacterium animalis subsp. *lactis* BLa80, a promising human probiotic strain isolated from breast milk of a healthy woman in Hongyuan, Sichuan Province, China. We have determined the complete genome sequence of strain BLa80, which consists of genes that are likely to provide useful information for its safe use as a probiotic in dietary supplements.

## ANNOUNCEMENT

*Bifidobacterium* has been used as one of the most commercially available probiotic organisms throughout the world in a variety of functional foods and infant formulas (1–4). The isolation of *Bifidobacterium* from breast milk is an attractive approach for the selection of a novel potential candidate of probiotics (5, 6). In this study, we isolated Bifidobacterium animalis subsp. *lactis* strain BLa80 from breast milk of a healthy woman in Hongyuan, Sichuan Province, China. Written informed consent was obtained from the volunteer, and all procedures for isolation and characterization were performed based on the principles of the Declaration of Helsinki.

Samples were diluted and 1 mL of dilution was incorporated into de Man-Rogosa-Sharpe medium agar, followed by anaerobic incubation at 37°C for 48 h. Single pure colonies were isolated and subcultured for morphological, biochemical, and molecular identifications. Based on 16S rRNA gene sequencing, strain BLa80 was identified as *B. animalis* subsp. *lactis*, which has been archived in the China General Microbiological Culture Collection Center with the number CGMCC NO.22547.

BLa80 was cultured anaerobically at 37°C for 18 h. Genomic DNA was extracted using a modified cetyltrimethylammonium bromide (CTAB) method (7). The quality and quantity of extracted DNA were examined using a NanoDrop 2000 spectrophotometer (Thermo Fisher Scientific, Waltham, MA, USA), a Qubit double-stranded DNA (dsDNA) high-sensitivity (HS) assay kit on a Qubit 3.0 fluorometer (Life Technologies, Carlsbad, CA, USA), and electrophoresis on a 0.8% agarose gel (8). High-quality genomic DNA was randomly fragmented, sheared into >10-kb fragments using a g-TUBE device, and then end repaired to prepare SMRTbell DNA template libraries. Whole-genome sequencing was performed by using the PacBio Sequel II platform at Frasergen Bioinformatics Co., Ltd., Wuhan, China.

A single SMRT cell produced a total of 5.26 Gb in 536,373 subreads (mean length, 9,810 bp). The *N*_50_ value of subreads was 10,946 bp, and all raw reads were processed using SMRTLink v10.1 (9, 10) with the parameter minlength set to 50 and all other parameters kept at default (https://www.pacb.com/support/software-downloads/). The genome assembly was performed using smrtlink v10.1 and function pb_assembly_microbial with the following parameters: microasm_coverage 30, microasm_genome_size 2000000, microasm_length_cutoff −1. The assembled contig was rotated and oriented to position the *dnaA* gene as the starting point in the complete genome by using an in-house Python script.

The genome of BLa80 was automatically annotated using the NCBI Prokaryotic Genomes Automatic Annotation Pipeline (PGAP) v5.3 (11). Functional annotation of predicted protein-coding sequences of BLa80 was conducted by utilizing Clusters of Orthologous Groups (COG), Kyoto Encyclopedia of Genes and Genomes (KEGG), and Gene Ontology (GO) databases.

The genome of *B. animalis* subsp. *lactis* BLa80 consists of a single circular chromosome with a length of 1,935,434 bp. The average genome coverage is 2,433.87-fold, without any plasmid or prophage sequence, and the G+C content of the genome is 60.5%. The genome contains a total of 1,550 putative protein-coding sequences (CDSs), along with 3 rRNA operons and 52 tRNA genes.

The strain is predicted to encode about 500 proteins involved in transport and metabolism of central carbohydrates and amino acids as well as cofactors and vitamins.

This work indicates that *B. animalis* subsp. *lactis* BLa80 has potential in adaption to environmental conditions. The complete genome sequence of BLa80 may be useful to better understand the probiotic properties of this strain and to provide additional insight into the genetic basis for survival and residence in the human gut.

### Data availability.

The complete genome sequences of *B. animalis* subsp. *lactis* BLa80 are available from NCBI GenBank under the accession number CP084116. The raw sequence reads have been deposited in the NCBI Sequence Read Archive (SRA; accession number SRR21276823) under BioProject accession number PRJNA758747 and BioSample accession number SAMN21499763.
